# Transcranial Doppler sonography follow-up study in mild vascular cognitive impairment

**DOI:** 10.1371/journal.pone.0317888

**Published:** 2025-01-24

**Authors:** Mariagiovanna Cantone, Manuela Pennisi, Giuseppe Lanza, Raffaele Ferri, Francesco Fisicaro, Francesco Cappellani, Emanuele David, Vito Nicosia, Klizia Cortese, Giovanni Pennisi, Valentina Puglisi, Rita Bella

**Affiliations:** 1 Neurology Unit, Policlinico University Hospital "G. Rodolico-San Marco", Catania, Italy; 2 Department of Biomedical and Biotechnological Sciences, University of Catania, Catania, Italy; 3 Department of Surgery and Medical-Surgical Specialties, University of Catania, Catania, Italy; 4 Clinical Neurophysiology Research Unit, Oasi Research Institute-IRCCS, Troina, Italy; 5 Ophthalmology Unit, Policlinico University Hospital "G. Rodolico-San Marco", Catania, Italy; 6 Department of Medical and Surgical Sciences and Advanced Technologies "G. F. Ingrassia", University of Catania, Catania, Italy; 7 Department of Educational Sciences, University of Catania, Catania, Italy; 8 Department of Neurology and Stroke Unit, ASST Cremona, Cremona, Italy; Icahn School of Medicine at Mount Sinai, UNITED STATES OF AMERICA

## Abstract

**Background:**

To date, few data to transcranial Doppler sonography (TCD) are available in patients with mild vascular cognitive impairment (VCI) at risk for vascular or mixed dementia. In a previous study in patients with mild VCI and cerebral small vessels disease, a hemodynamic pattern of cerebral hypoperfusion and enhanced vascular resistance were observed; however, longitudinal data are currently lacking. Here, we perform a clinical, psychopathological, and neurosonological follow-up of patients with VCI in order to monitor any progression and to identify TCD measures to detect it.

**Methods:**

From the original cohort of 161 patients, 127 with VCI (mean age 73.6 ± 7.1; 67 males) were re-evaluated after 5.0 ± 1.8 years. Namely, the Montreal Cognitive Assessment (MoCA), the 17-items Hamilton Depression Rating Scale (HDRS), and the Stroop Color-Word Interference Test (StroopT) were administered to screen for global cognitive status, to quantify depressive symptoms, and to explore executive functions, respectively. Mean blood flow velocity (MBFV), peak systolic blood flow velocity (PSV), end-diastolic blood flow velocity (EDV), pulsatility index (PI), and resistivity index (RI) were recorded from the middle cerebral artery, bilaterally.

**Results:**

At follow up, patients exhibited a significant worsening of both MoCA (21.7 ± 2.1 *vs*. 20.7 ± 2.0) and StroopT scores (57.4 ± 19.4 *vs*. 59.7 ± 18.6), whereas HDRS showed an improvement, although the mean raw score remained above the cut-off value for depression (10.3 ± 6.6 *vs*. 9.8 ± 6.3). MBFV, PSV, and EDV showed a significant increase in PSV and PI and a reduction in EDV. When focused to younger patients (<65 years), we confirmed the significant worsening of both MoCA and StroopT but not HDRS, as well as the significant changes in PI and RI. Finally, considering the differences (D) between baseline and follow-up, the following significant correlations emerged, although with a small-to-medium effect size for all of them: positive correlation between MBFV-D and MoCA-D and between RI-D and STROOP-D, and a negative significant correlation between RI-D and MoCA-D.

**Conclusions:**

Notwithstanding some limitations, such as the lack of a control group and neuroimaging data at follow-up, TCD may contribute to the early detection, monitoring, and management of VCI patients at risk for dementia. Together with compatible clinical and cognitive features, the exploration of early TCD markers that possibly indicate a higher risk of progression might represent an intriguing research direction and a significant clinical perspective.

## Introduction

Small vessel disease (SVD) is a multifaceted disorder of cerebral microvessels causing different types of neuropathological lesions that are commonly seen at brain imaging, especially in older subjects. Typically, these include white matter hyperintensities, lacunes, microbleeds, superficial siderosis, enlarged perivascular spaces, and microinfarcts [1]. However, other changes are also detected through more sensitive magnetic resonance imaging (MRI) methods. These lesions can affect the so-called “normal appearing white matter” and even the grey matter, with cortical atrophy and other signs of degeneration. Globally, they are likely due to the disruption of the white matter tracts connecting cortical and subcortical areas (“disconnection” hypothesis) [2].

These neuroradiological findings may have a variety of presentations, ranging from clinically silent to overt symptomatic lesions, variably followed by different cognitive, behavioral, and mood consequences, being the executive dysfunction, apathy, and depression, respectively, the most common manifestations of these patients [3]. In this context, the impact of cerebral SVD is greatly recognized by its role in the onset and progression of vascular cognitive impairment (VCI) [4]. As a whole, VCI is now viewed as the most common cause of cognitive decline in the elderly and a significant contribution (up to 50%) also in the development of Alzheimer’s disease (AD) [5].

In this scenario, transcranial Doppler sonography (TCD) is a non-invasive, cost-effective, and feasible tool, readily accessible for routine clinical use, that employes a low-frequency ultrasound probe to study cerebral blood velocity in the basal cerebral arteries [6–8]. The middle cerebral artery (MCA) and the basilar artery (BA) are the most studied for the neurosonological assessment of the anterior and posterior vascular supply and hemodynamics, respectively [9]. Briefly, TCD allows monitoring of blood flow velocity and measures of downstream resistance in blood flow, mainly through the resistivity index (RI) and the pulsatility index (PI), with high temporal resolution [10].

TCD emerges also as a useful investigation in the assessment of dementia and cognitive impairment [11], also providing intriguing research hints. In particular, the practical utility and relevance of TCD in patients’ management is indexed by alterations of cerebrovascular hemodynamics in normal aging, mild cognitive impairment (MCI), and dementia, as well as in different etiologies of dementia, as recently reported in a systematic review and meta-analysis of TCD studies [12]. Among them, in a recent cross-sectional study, we have investigated the role of cerebral hemodynamics in elderly patients with mild VCI compared to those with vascular white matter lesions (WMLs) but without any cognitive deficit [13]. Shortly, patients showed a hemodynamic pattern indicative of cerebral hypoperfusion and enhanced vascular resistance, that may be considered as the TCD correlate of mild VCI due to SVD. Translationally, TCD was able to provide indices of the occurrence and severity of microcirculation pathology, which correlated with executive dysfunction [13]. A follow-up would allow a more comprehensive understanding of the complex relationships between hemodynamic changes, WMLs, and risk of dementia. Indeed, the possibility to early detect any marker of transition into overt dementia is needed to support clinicians towards a more careful diagnosis and management of this population at risk. However, to date, very few TCD data are available in patients with mild VCI and longitudinal data in this population of subjects at risk for vascular or mixed dementia are currently lacking. This is a gap in the literature, probably due to the difficulty of selecting homogeneous samples of patients and following them with a long-lasting follow-up. To fill this gap, longitudinal studies on large samples, with a multidimensional approach and clear objective outcome measures, should be encouraged.

In this longitudinal study, we aim to monitor the indexes of cerebral hemodynamics in patients with mild VCI after a follow-up period of approximately 5 years, in order to detect any TCD marker of disease progression into dementia. We hypothesize that, according to the natural history of VCI, the follow-up evaluation of these subjects would show a worsening also at the level of TCD parameters, thus representing the neurosonological correlates of cognitive decline.

## Materials and methods

### Study design

This is a prospective study on mild VCI patients previously enrolled and identified according to the International Society for Vascular Behavioral and Cognitive Disorders criteria [14]. Patients underwent clinical, psycho-cognitive, and neurosonological re-evaluation after 5.0 ± 1.8 years. Those subjects with WMLs but without any cognitive deficit, who served as a control group in the previous study, were not re-evaluated in the present investigation.

### Subjects’ assessment

All subjects were clinically re-evaluated and assessed for vascular risk factors, as well as for any antihypertensive, antiplatelet, anticoagulant, lipid-lowering, hypoglycemic drugs, and antidepressant drug taken. Then, they underwent the same neuropsychological assessment as at entry, which included: the Montreal Cognitive Assessment (MoCA), a multidimensional cognitive screening test evaluating different cognitive domains [15]; the Stroop Color-Word Interference Test (StroopT), a sensitive measure of executive functions, namely attention and response inhibition to conflicting stimuli [16] (normative values collected from an Italian population sample, StroopT score ≤ 36.92 s [17]); the 17-items Hamilton Depression Rating Scale (HDRS), an internationally validated scale for the quantification of depressive symptoms [18].

Exclusion criteria were: history of stroke/transient ischemic attack or other neurological disorders (e.g., Parkinson’s disease, head trauma, epilepsy); major psychiatric disorders (e.g., schizophrenia, obsessive-compulsive disorder, depressive disorders); any uncompensated acute or chronic medical illness; endocrinopathies affecting cognitive functions; alcohol or drug abuse; ultrasound evidence of carotid or vertebral extracranial artery stenosis ≥ 50% prior to the enrolment; ultrasound evidence of intracranial artery stenosis; bilateral absence of adequate transtemporal windows for TCD examination.

This study was carried out in accordance with the Declaration of Helsinki of 1964 and its later amendments. The protocol was approved by the Ethics Committee of the “Azienda Ospedaliero-Universitaria “Policlinico—Vittorio Emanuele” of Catania, Italy (approval code: prot. N. 41591; approval date: 27/09/2018). All subjects gave written informed consent prior to entry and all procedures respected the standards of good clinical practices. The start and end of the recruitment period for this study were January 2023 and July 2024, respectively.

### Transcranial Doppler

A detailed description of TCD methodology was previously reported [13]. Briefly, TCD was performed with Compumedics DWL equipment, Multi-Dop X digital, Singen (Germany). All the examinations were performed by the same operator (R.B.), who remained “blind” with respect to the patients’ neuropsychological performance. Velocimetric parameters were obtained bilaterally from the M1 segment of the MCA, bilaterally, which was insonated through the transtemporal window using a 2 MHz DWL probe at a depth of 50–60 mm.

The following TCD parameters were obtained and recorded: peak systolic velocity (PSV); end-diastolic velocity (EDV); mean flow velocity (MBFV); PI, derived from the Gosling and King formula: (PSV-EDV)/MBFV; RI, derived from the Pourcelot formula: (PSV-EDV)/PSV. All parameters were acquired after 30 seconds of stable recording and for at least 10 consecutive cardiac cycles. Data were the average of the two measurements, one for each side; otherwise, the measurement from the only available side was used. Blood pressure and heart rate were recorded before each examination. Data were collected on a dedicated PC and stored in an ad hoc database.

### Statistical analysis

Between-group comparisons of clinical, neuropsychological, and neurosonological parameters were carried out by means of the Student’s *t*-test, associated to the computation of the Cohen’s *d* effect size. According to Cohen, effect sizes can be classified as small (*d* = 0.2), medium (*d* = 0.5), and large (*d* ≥ 0.8). Correlations between neuropsychological and TCD variables were evaluated by means of Pearson’s correlation coefficient; following the Cohen’s [19] indications, we considered a correlation coefficient 0.10, 0.30, and 0.50 as corresponding to small, medium, and large sizes, respectively. A *p* value lower than 0.05 was considered as statistically significant.

## Results

From the baseline cohort of 161 patients, 34 were lost to follow-up because they died or could not be reached anymore. Therefore, the follow-up study was carried out on a total convenience sample of 127 VCI patients (60 females and 67 males, aged 74.00 ± 6.85 and 73.30 ± 7.28 years respectively, *p* = 0.424). With this sample size, we obtained a power of 80% to detect a significant difference (alpha 0.05) with a moderate effect size of 0.5. As a whole, at the follow-up visit, all participants had a mean age of 73.60 ± 7.07 years, a mean education of 6.90 ± 3.67 years, and a mean score at the Mini Mental State Examination (MMSE) of 25.30 ± 1.39 (normal value, adjusted for age and education, ≥ 24).

As shown in Table 1, which compares neuropsychological tests and TCD parameters between baseline (T0) and follow-up (T1), some key, statistically significant, findings emerged for all the neuropsychological variables considered, namely: MoCA dropped and StroopT increased; in terms of raw score, HDRS improved, although it remained above the cut-off value for mild depression. Similarly, TCD parameters showed a statistically significant increase in PSV and a reduction in EDV; the same holds true for PI and RI.

**Table 1:** Comparison between baseline (T0) and follow up (T1) of neuropsychological and TCD variables in the whole group of patients with vascular cognitive impairment (VCI).

Variable	Baseline (T0)		Follow-up (T1)		Student’s *t*-test		Cohen’s *d*
	Mean	SD	Mean	SD	*t*	*p <*	*Effect size*
**MoCA**	21.65	2.087	20.67	1.948	12.211	0.000001	0.495
**HDRS**	10.27	6.615	9.80	6.302	3.893	0.00016	0.077
**StroopT**	57.42	19.390	59.68	18.633	-8.430	0.000001	-0.121
**MBFV**	52.64	5.651	51.96	5.387	3.446	0.0008	0.109
**PI**	0.94	0.141	1.04	0.182	-7.255	0.000001	-0.620
**PSV**	85.58	9.638	87.84	8.748	-5.268	0.000001	-0.239
**EDV**	36.30	4.586	34.02	5.603	7.566	0.000001	0.449
**RI**	0.58	0.055	0.61	0.064	-7.143	0.000001	0.000

MoCA = Montreal Cognitive Assessment; HDRS = Hamilton Depression Rating Scale; StroopT = Stroop Color Word Interference Test (seconds); MBFV = mean blood flow velocity (cm/s); PI = pulsatility index; PSV = peak systolic velocity (cm/s); EDV = end-diastolic velocity (cm/s); RI = resistivity index; SD = standard deviation.

Notably, when restricted to younger patients, i.e., < 65 years (Table 2), the results revealed a significant worsening of both MoCA and the StroopT scores but not for HDRS, and significant changes in PI and RI but not for the other TCD parameters. The same comparison in patients aged ≥ 65 years (Table 3) yielded results very similar to those reported in Table 1.

**Table 2:** Comparison between baseline (T0) and follow up (T1) of neuropsychological and TCD variables in the group of patients with vascular cognitive impairment (VCI) aged < 65 years.

Variable	Baseline (T0)		Follow-up (T1)		Student’s *t*-test		Cohen’s *d*
	Mean	SD	Mean	SD	*t*	*p <*	*Effect size*
**MoCA**	21.89	2.470	20.95	2.527	4.869	0.00012	0.400
**HDRS**	10.16	7.904	9.79	7.700	1.197	0.247	0.051
**StroopT**	58.92	20.208	60.49	19.329	-3.333	0.0037	-0.081
**MBFV**	53.37	2.891	53.47	3.025	-0.233	0.818	-0.034
**PI**	0.94	0.145	1.01	0.157	-3.060	0.0067	-0.665
**PSV**	86.99	6.574	89.47	6.168	-2.060	0.054	-0.392
**EDV**	36.56	3.965	35.47	3.921	1.876	0.077	0.279
**RI**	0.57	0.053	0.60	0.055	-2.933	0.0089	0.000

MoCA = Montreal Cognitive Assessment; HDRS = Hamilton Depression Rating Scale; StroopT = Stroop Color Word Interference Test (seconds); MBFV = mean blood flow velocity (cm/s); PI = pulsatility index; PSV = peak systolic velocity (cm/s); EDV = end-diastolic velocity (cm/s); RI = resistivity index; SD = standard deviation; NS = not significant.

**Table 3:** Comparison between baseline (T0) and follow up (T1) of neuropsychological and TCD variables in the group of patients with vascular cognitive impairment (VCI) aged ≥ 65 years.

Variable	Baseline (T0)		Follow-up (T1)		Student’s *t*-test		Cohen’s *d*
	Mean	SD	Mean	SD	*t*	*p <*	*Effect size*
**MoCA**	21.61	2.022	20.62	1.838	11.165	0.000001	0.513
**HDRS**	10.29	6.404	9.80	6.066	3.703	0.00034	0.079
**StroopT**	57.15	19.328	59.53	18.597	-7.850	0.000001	-0.126
**MBFV**	52.51	6.007	51.69	5.671	3.789	0.00025	0.139
**PI**	0.94	0.141	1.05	0.186	-6.680	0.000001	-0.646
**PSV**	85.34	10.084	87.56	9.119	-4.826	0.000005	-0.231
**EDV**	36.25	4.702	33.76	5.826	7.405	0.000001	0.469
**RI**	0.58	0.056	0.61	0.066	-6.526	0.000001	-0.588

MoCA = Montreal Cognitive Assessment; HDRS = Hamilton Depression Rating Scale; StroopT = Stroop Color Word Interference Test (seconds); MBFV = mean blood flow velocity (cm/s); PI = pulsatility index; PSV = peak systolic velocity (cm/s); EDV = end-diastolic velocity (cm/s); RI = resistivity index; SD = standard deviation; NS = not significant.

Regarding sex, Table 4 shows no statistically significant difference between males and females for any neuropsychological or TCD variable considered, except however for the HDRS score, which was higher in females.

**Table 4:** Baseline (1)–follow up (2) comparison between male and female VCI patients.

Variable	Females		Males		Student’s *t*-test		Cohen’s *d*
	Mean	SD	Mean	SD	*t*	*p <*	*Effect size*
**MoCA-1**	21.38	1.992	21.90	2.154	-1.386	0.168	-0.241
**HDRS-1**	11.58	6.758	9.09	6.302	2.151	0.033	0.383
**StroopT-1**	58.43	19.407	56.51	19.476	0.555	0.580	0.098
**MBFV-1**	52.73	5.474	52.55	5.845	0.180	0.858	0.018
**PI-1**	0.95	0.132	0.94	0.149	0.498	0.619	0.000
**PSV-1**	86.17	8.851	85.06	10.330	0.644	0.521	0.114
**EDV-1**	36.28	4.103	36.31	5.010	-0.026	0.979	0.000
**RI-1**	0.58	0.063	0.57	0.046	1.501	0.136	0.000
**MoCA-2**	20.32	1.712	20.99	2.100	-1.952	0.053	-0.366
**HDRS-2**	11.08	6.336	8.64	6.089	2.213	0.029	0.402
**STROOP-2**	60.74	18.897	58.73	18.484	0.606	0.546	0.107
**MBFV-2**	51.87	5.432	52.04	5.386	-0.185	0.853	-0.018
**PI-2**	1.05	0.168	1.04	0.194	0.532	0.595	0.555
**PSV-2**	88.02	8.574	87.69	8.963	0.211	0.833	0.034
**EDV-2**	33.79	5.412	34.22	5.802	-0.433	0.666	-0.071
**RI-2**	0.61	0.058	0.61	0.070	0.652	0.516	0.000

MoCA = Montreal Cognitive Assessment; HDRS = Hamilton Depression Rating Scale; StroopT = Stroop Color Word Interference Test (seconds); MBFV = mean blood flow velocity (cm/s); PI = pulsatility index; PSV = peak systolic velocity (cm/s); EDV = end-diastolic velocity (cm/s); RI = resistivity index; SD = standard deviation; NS = not significant.

**Fig 1:**
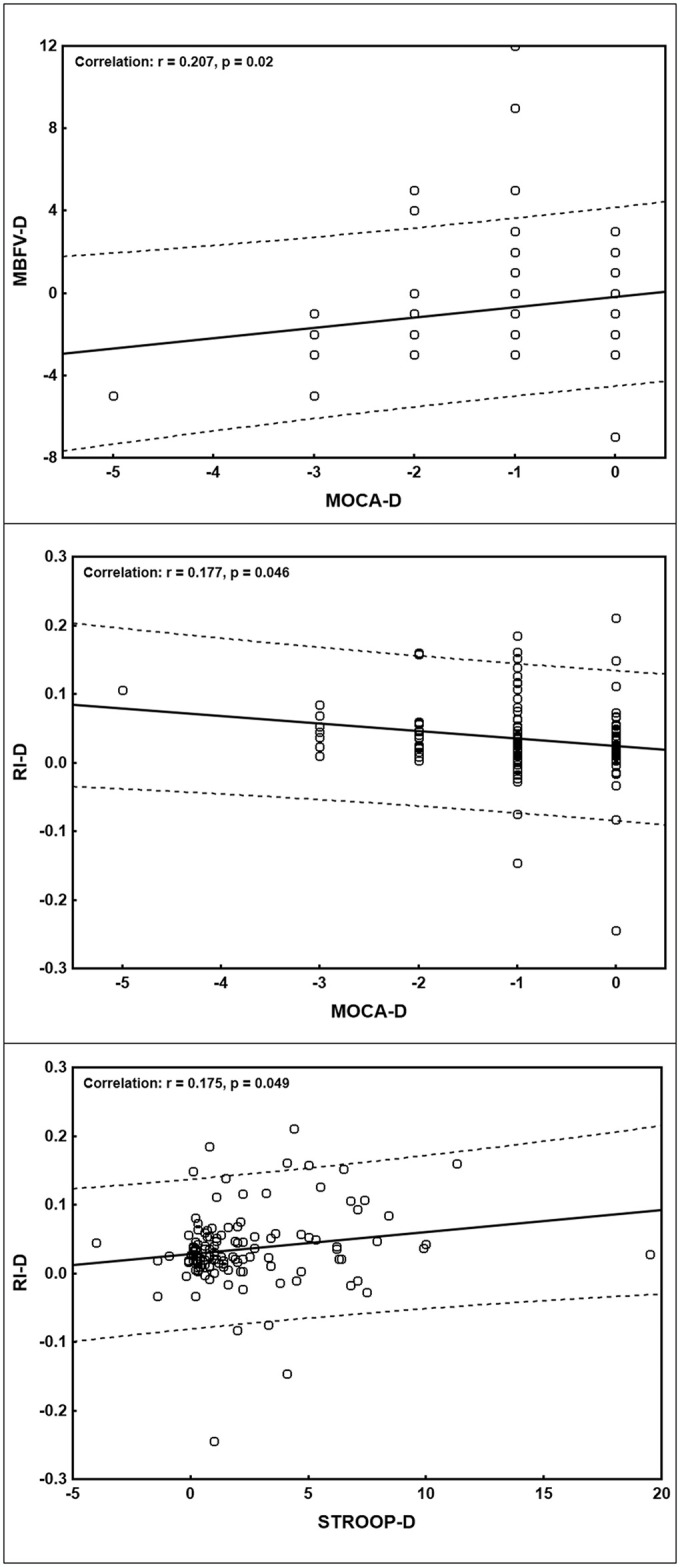
Correlations differences (delta, D) between baseline (T0) and follow-up (T1) of Montreal Cognitive Assessment (MoCA) and mean blood flow velocity (MBFV, top panel), MoCA and resistivity index (RI, middle panel), and STROOP and RI (bottom panel). The regression line is also shown (continuous line) in each panel, as well as the 95% prediction lines (dashed lines) indicating the range within which 95% of new observations are expected.

Finally, the correlation analyses of the differences in terms of delta (D) between T0 and T1 in neuropsychological variables (MoCA-D, HDRS-D, StroopT-D) and TCD parameters (PI-D, PSV -D, EDV-D, RI-D, MBFV-D), disclosed some additional relevant findings, i.e., a positive statistically significant correlation between MBFV-D and MoCA-D and between RI-D and STROOP-D, and a negative significant correlation between RI-D and MoCA-D (Fig 1). However, all these correlations were “small-to-medium”, following Cohen’s recommendations [19].

## Discussion

### Main findings

To our knowledge, this is the first follow-up study exploring hemodynamic changes to TCD in mild VCI. Compared to the baseline, these patients demonstrated a significant worsening of the MBFV, likely reflecting a pattern of hypoperfusion due to cerebral SVD [20]. Results also showed an increase in PSV and a decrease in EDV; although these hemodynamic changes might contribute to the understanding of processes underling VCI [12, 21], it cannot be excluded that the same alterations could parallel an accelerated bran aging or be the consequence of reduced cellular metabolism due to progressive cerebral atrophy [11, 22, 23]. Indeed, the distinction between normal aging and pathological changes in TCD findings remains unclear. However, while aging affects cerebral hemodynamics [24], the observed correlations between TCD parameters and cognitive decline in our study suggest an accelerated pathological process in VCI [25]. Nevertheless, future studies with age-matched controls and advanced imaging are needed to clarify this differentiation. Similarly, both PI and RI, typically considered as markers of peripheral vascular resistance, increased significantly. This is a finding possibly due to the progression of vascular alterations underling SVD, along with structural remodeling, loss of vasomotor reactivity, and impaired neurovascular coupling [25]. Overall, the main translational implication is represented by the potential role of TCD in identifying hemodynamic markers for risk of dementia.

In particular, PI, which showed the largest increase, is a very accurate index in the identification of altered hemodynamics in subjects with cerebrovascular disease. PI in cognitive impairment is particularly useful in defining its severity and in differentiating between vascular and degenerative forms of cognitive decline [12]. It is known that PI increases when the systemic pulse pressure delivered to M1 increases. However, PI is not a mere measure of vascular resistance, as it also correlates with arterial compliance, heart rate, pulse amplitude, cerebral perfusion pressure, and mean arterial pressure [12, 26]. Therefore, the increase in PI observed in our cohort of VCI patients may reflect a compensatory increase in cerebral perfusion pressure to contrast the reduction of MBFV, thus counteracting the effect of microvascular structural alterations [20, 26–28].

Regarding RI, changes usually indicate alterations in intracranial neurovascular flow dynamics, which may include variations in blood volume, flow velocity, and vascular resistance. It is reported that this parameter is impaired in perinatal asphyxia with damage of the white matter, also with a predictive role of injuries [29–31], in vascular parkinsonism with a high grade of leukoaraiosis [32], and in patients with metabolic disorders such as cirrhosis with hyperdynamic circulatory state [33]. In the present study, the increase of RI was independently associated with cognitive worsening, thus suggesting a role in the worsening of cognitive decline and a possible use of TCD in clinical practice to predict long-term monitor of the progression of cerebrovascular disease [34].

Taken together, the time course of these hemodynamic parameters appears consistent with the baseline TCD pattern observed in our patients, in which a reduction in MBFV and an increase in both PI and RI compared to controls were suggested [13]. Translationally, although the lack of control group and neuroimaging data in the present study, which preclude a fully assessment of age-related changes and longitudinal TCD-MRI correlations, respectively, these findings confirm the chronic and progressive course of VCI. Concomitantly, they emphasize the role of TCD in the follow-up, prognosis, and management of these patients. Similar results are reported in the literature, although limited to VaD, AD, and MCI. A recent meta-analysis confirms the reduction of MBFV in all patients with cognitive impairment, but the increase in PI was observed only in those with overt dementia [12]. Of note, another meta-analysis highlighted similar cerebral hemodynamic findings, although the decline in MBFV was more pronounced in subjects with a vascular form of cognitive decline [35]. In this context, a number of novel translational approaches to study cerebrovascular pathology have been developed over time, including retinal vascular analyses [36–38].

A result of particular interest emerges from the stratification of patients by age, since the younger subjects (< 65 age) are at higher risk of future dementia and death, also given their potential long course of disease [39]. In this age group, we found a significant worsening of both PI and RI, but not of velocity parameters. This is probably due to the greater sensitivity and early modification of these parameters in younger VCI patients at risk for progression. It the same way, it is likely that the increase in resistance will cause variations in the velocity parameters at a later stage, when the reduction in vascular compliance and the increase in stiffness of the arterial wall become an evident consequence of vascular degeneration. Therefore, this index might be proposed as a screening tool for cognitive decline and a target for preventive strategies and disease-modifying therapies [20, 40].

Cognitively, the worsening of the mean MoCA and StroopT score at follow-up are the main neuropsychological findings, confirming a global clinical worsening of these patients, and of executive functions in particular, which are widely evaluated through the tests used [15, 16]. The substantial and progressive impairment of this cognitive domain, not paralleled by a consensual decrease of the mean MMSE score [41], falls within the typical clinical presentation of VCI [42–44]. Therefore, we confirm general recommendation in favor of MoCA, rather than MMSE, for screening and monitoring both vascular and degenerative forms of cognitive impairment [45].

Finally, a relevant result concerns changes in depressive symptoms. We observed a slight, but statistically significant, improvement in HDRS score, despite it remained above the threshold for depression. Depressive symptoms are peculiar elements of the clinical picture of VCI patients, which are not only an indirect effect of disability and impaired quality of life, but also a direct consequence of disruption of specific neurobiological networks (mostly located at fronto-striato-limbic regions), responsible for mood and affect regulation [46]. The improvement in HDRS score might be due to the possibility that these patients, over time, have progressively acquired greater awareness and tolerance of their cognitive deficits, with some of them partially recovering spontaneously or thanks to the implementation of coping strategies (i.e., active management and adaptation to stress) [47].

From a pure demographic perspective, age did not seem to be a particularly influential factor in the development and course of depression in patients with VCI. Indeed, a non-significant change in the HDRS score in patients under 65 years was noted, unlike the significant worsening of both MoCA and StroopT scores. However, the possibility that this non-significant change might related to the relatively small sample size in this subpopulation cannot be excluded. Conversely, according to the stratification by sex, the HDRS score was the only but relevant parameter showing a statistically significant difference in females at both T0 and T1. This is in line with epidemiological data identifying depression as twice common in females than in males [48]. This phenomenon, known as "depression gender gap", arises from a wide neurobiological scenario (i.e., genetic background, hormonal factors, and response to stress) and a psychological predisposition, both linked to a higher susceptibility to environmental factors [49], along with complex neurophysiological substrates [50].

Finally, the negative correlation between RI-D and MoCA-D and the positive correlation between the same TCD parameter and StroopT-D highlight the significant relationship between cognitive worsening, progression of executive disfunction, and increased vascular resistance. Although these correlations were “small-to-medium”, they suggest a possible role of TCD in the long-term monitoring of progression of the cerebrovascular-related damage in these patients [34].

### Strengths and limitations

Some strengths and limitations of this study should be acknowledged. The main advantage are the longitudinal approach and the stratification for age and sex of these patients. Moreover, despite some drop-out, the cohort remained sufficiently large and representative of VCI, thus reasonably allowing a generalization of the results obtained.

Regarding limitations, apart from the relatively small sample size, the main is the lack of a control group at follow-up, who did not have any cognitive deficit at baseline but who could have developed it (at least some of them) at follow-up, having this allowing a more comprehensive comparison with the group of subjects re-evaluated here. A second limitation is that MRI was not repeated at follow-up and, therefore, a TCD-MRI correlation remains a future avenue of research. An intriguing opportunity would be the implementation of TCD evaluation with additional parameters, such as those exploring the vasomotor reactivity, which is accurate in identifying VCI and in discriminating between subjects with or without dementia [11, 26]. Similarly, the integration of MRI data, also deriving from advanced techniques (such as those for the study of the so-called “normal appearing white matter”) will allow a better clinical-radiological correlation of these patients, both cross-sectionally and longitudinally, in order to more accurately stratify the risk of progression and early identify those with dementia. The same applies to the use of a broader battery of neuropsychological tests, capable of revealing even subtle deficits in different cognitive domains, to be put into correlation with clinical, neurosonological, and imaging data. Lastly, the lack of tools allowing a definitive differential diagnosis between pure and mixed forms of VaD can lead, at least in some cases, to an overestimation of the vascular contribution [12].

## Conclusions

Hemodynamic dysfunction may play a pathogenic role in the development and progression of cognitive impairment in elderly patients with SVD. Although further validation is needed before a widespread clinical application, the exploration of early TCD markers that possibly indicate a higher risk of progression represent an intriguing research direction and a significant clinical perspective.

## Supporting information

S1 Dataset (XLSX)
